# Poorly Investigated Ecuadorian Medicinal Plants

**DOI:** 10.3390/plants11121590

**Published:** 2022-06-16

**Authors:** Chabaco Armijos, Jorge Ramírez, Giovanni Vidari

**Affiliations:** 1 Departamento de Química, Universidad Técnica Particular de Loja, Loja 1101608, Ecuador; jyramirez@utpl.edu.ec; 2 Department of Medical Analysis, Faculty of Applied Science, Tishk International University, Erbil 44001, Iraq; vidari@unipv.it

**Keywords:** medicinal plants, Ecuador, traditional uses, ethnomedicine, ethnobotany

## Abstract

Ecuador has, in proportion of its size, one of the richest floras of Latin America and the world; the country also has an immense cultural heritage due to the presence of different ethnic groups that have implemented the use of many wild and cultivated plants, mainly as medicinal remedies. In a recent publication, we have summarized the results of research activities recently carried out on about 120 plants native to Ecuador, which includes the structures of non-volatile isolated compounds, as well as the chemical composition of essential oils (EOs) and the in vitro tested biological activity data. For the sake of completeness, we have collected in this paper the main information obtained from recent ethnobotanical investigations on other important Ecuadorian medicinal plants for which phytochemical, pharmacological, and toxicological studies are, however, still largely lacking. Thus, one of the objectives of this paper is to preserve the traditional knowledge of Ecuadorian Indigenous communities which, being transmitted orally, is in danger of becoming lost. Moreover, it is our intention to stimulate more extensive studies on the rich medicinal flora of the country, which can provide economic and social benefits, especially to the people who traditionally cultivate or collect the plants.

## 1. Introduction

The different ethnic groups living in Ecuador have managed, through a process of social and biological evolution, to manage and use numerous medicinal plants for their own benefit [1,2]. In fact, in Indigenous communities, traditional healer practitioners and shamans use a variety of medicinal plants and natural remedies for their health-care practices and religious rituals. This knowledge is entirely empirical and is passed orally from generation to generation, thus it is in danger of becoming lost [3]. Moreover, the ancestral consumption of medicinal and hallucinogenic plants by native peoples [4] is based on popular traditions, and on the apparent efficacy and safety of these remedies for the treatments and cures of ailments of different kinds, or for performing rituals [5]. However, no scientific evidence has validated most of these effects. This situation is common to most developing countries, where the cost of western world drugs is often prohibitive. As a result, it is estimated that about 60% of the world’s population uses plants almost exclusively as a source of medicines, although there is generally no control over the chemical compositions, while the effectiveness and the absence of chronic toxicity are all to be demonstrated.

**Figure 1:**
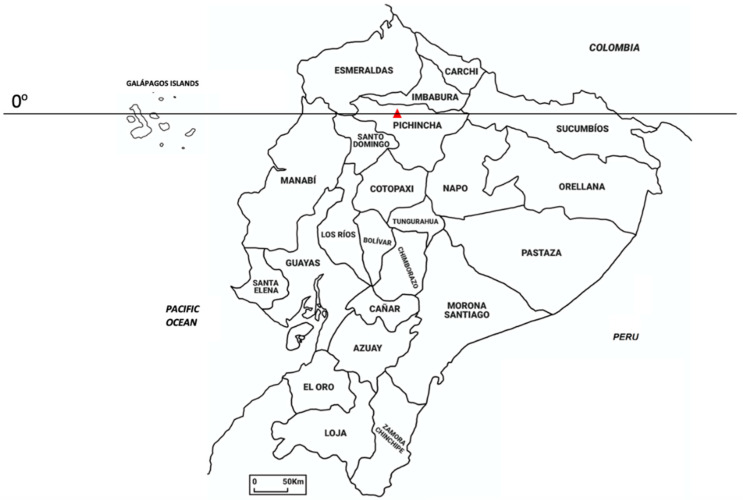
Provinces of Ecuador.

Ecuador (Figure 1), thanks to its high biodiversity, is included in the list of the 17 megadiverse world countries [6], all of which are partially or totally located between the tropics. Moreover, it is worthwhile to note that the surface of Ecuador is the smallest among the megadiverse countries, with around 258,000 km^2^, which correspond to only 0.02% of the earth’s surface. Regarding the number of native vascular plants growing in Ecuador, the *Catalogue of the Vascular Plants of Ecuador*, published in 1999, listed a total of 15,306 species, including 1298 pteridophytes, 17 gymnosperms, and 13,991 angiosperms [7]. Recently, the number of native taxa has been increased to 17,500 [8,9], of which from 4500 to 5500 are endemic [10,11]. However, it is estimated that, with the assessment of the studies on the Ecuadorian flora, the total number of vascular plants could reach 25,000 [11], which is just below 7% of the world’s known plants.

Despite the worldwide recognition as a megadiverse country, there is no general agreement on which components of Ecuador’s biodiversity are to be studied scientifically and used sustainably as a priority. In this context, we believe that an intelligent exploitation of the different useful vascular plants, and within them the hundreds of medicinal species used by the Indigenous communities, could provide numerous economic and social benefits to the entire population of Ecuador. In this regard, it is important to remember that the World Health Organization (WHO) has recommended the studies in the field of traditional medicinal plants as an aid to developing countries [12]. It should also be considered that more than 25% of the drugs on the market in industrialized countries are based on natural compounds or their derivatives; in particular, 60–80% of antibacterial and anticancer drugs are of natural origin [13,14]. Thus, the search for new bioactive lead compounds of natural origin, especially from poorly investigated regions of biodiversity, remains one of the main strategic lines of pharmaceutical research.

For these reasons, aimed at enhancing the value of the botanical resources of Ecuador and at sustaining the medicinal uses of local plants by scientific evidence, we have summarized the results of research activities carried out on hundreds of species growing in Ecuador in a couple of recent publications [2,15]. They include the structures of isolated non-volatile compounds, as well as the chemical composition of essential oils (EOs) and in vitro tested biological activity data. However, phytochemical, pharmacological, and toxicological studies are still largely lacking for several other native and endemic plants used in the traditional medicine of Ecuador, which are mentioned in a few ethnobotanical studies [15,16,17,18,19,20,21,22]. We believe that also orphan or poorly investigated medicinal plants may become important sources of secondary biologically active metabolites and give different opportunities for their sustainable uses. Therefore, in this paper, we have critically collected the available pertinent information about poorly investigated traditional native and endemic plants of Ecuador (Table 1), with the intention to stimulate further scientific investigations on the rich medicinal flora of the country. When reported in literature, phytochemical and/or pharmacological data of a species included in Table 1 are briefly described. For the sake of ethnopharmacological completeness, imported species used in the traditional medicine of Ecuador, which have not been reported in the previous reviews [2,15], are included in Table 2.

## 2. Research Strategies and Literature Sources

The data included in this paper have been retrieved using the keywords “medicinal plants from Ecuador”, “ethnomedicine”, “traditional uses”, and “medicinal plants” in different databases including PubMed, SciFinder, Springer, Elsevier, Wiley, Web of Science, and Google Scholar.

Plants with incorrect botanical classification or without the name of the species have not been included in Table 1. The plant scientific names were checked with the database WFO (2021): World Flora Online, published on the Internet at http://www.worldfloraonline.org [23] (accessed on 23 December 2021); Tropicos.org. Missouri Botanical Garden at https://www.tropicos.org/home [24] (accessed on 28 December 2021); Global Biodiversity Information Facility Ecuador at https://www.gbif.org/es/country/EC/summary [25] (accessed on 29 December 2021), and *Enciclopedia de las Plantas Utiles del Ecuador* [8]. Information from master’s and doctoral dissertations were not considered for the preparation of this review.

## 3. Ethnobotanical and Ethnopharmacological Data

A total of 257 medicinal plants are listed in Table 1 and Table 2, divided by 78 botanical families. They include 141 native and 11 endemic species (Table 1), and 105 species introduced from different regions of the world (Table 2). For each taxon appearing in the Tables, the botanical and the common names, the used part(s), as well as the traditional uses, are also reported.

The botanical family with the highest number of medicinal plants listed in Table 1 and Table 2 is Asteraceae (10.5%) with 15 native, 1 endemic, and 11 introduced species, followed by Lamiaceae (5.8%) with 4 native and 11 introduced species, and Solanaceae (5.1%) with 13 native species. The other botanical families contain from 1 to 11 species, accounting from 0.4 to 4.3% of the total number of taxa.

The use of endemic and native medicinal species exceeds by far the use of introduced medicinal species. On the other hand, introduced plants have an extensive use in the traditional medicine of Ecuador. This finding has been explained by the great abundance or accessibility (availability hypothesis), the ability to cure pathological conditions that are not treated by native plants (diversification hypothesis), or as a result of many different simultaneous uses (versatility hypothesis) [26].

**Table 1:** Botanical and vernacular names, phytochemical and pharmacological data, used part(s), and traditional uses of native and endemic medicinal plants of Ecuador ^a^.

No.	Botanical Name	Vernacular Name	Phytochemical and Pharmacological Data ^b^	Used Part(s) of the Plant	Traditional Uses
	**ACANTHACEAE**				
1	*Justicia pectoralis* Jacq.	*Saucillo*, *tigrecillo*	No information is reported in literature.	Branches	It is used orally to treat general disorders of nervous and dermatological systems, and culture-related syndromes [4,16,20,21,22].
	**AMARANTHACEAE**				
2	*Alternanthera porrigens* (Jacq.) Kunze	*Moradilla*	No information is reported in literature.	Branches, flowers	It is used orally and in baths to treat general disorders of the gynecological system [20,21].
3	*Amaranthus caudatus* L.	*Amaranto*, *ataco morado*	No information is reported in literature.	Inflorescences	It is used orally and in baths to treat disorders of the circulatory, gynecological, and respiratory systems [20,21].
4	*Amaranthus cruentus* L.	*Ataco*, *sangorache*	No information is reported in literature.	Inflorescences, leaves	Anti-inflammatory, astringent, anti-flu, antihemorrhagic, diuretic and tonic, carminative, emmenagogue, hepatic, stimulant, to enhance blood circulation, and to treat abdominal pain related to menstruation [4,16,20].
5	*Amaranthus hybridus* L.	*Bledo*, *ataco*	No information is reported in literature.	Inflorescences	It is used orally to treat general disorders of the circulatory, gynecological, respiratory, and urinary systems [21].
6	*Amaranthus quitensis* Kunth	*Ataco*	No information is reported in literature.	Leaves, roots	Pain relief (at joints, head, throat), and to treat gastrointestinal and respiratory problems [18].
7	*Iresine diffusa* Humb. & Bonp. Ex Willd	*Tigrecillo*, *velo de novia*, *chulco*, *escancel*	No information is reported in literature.	Branches	In topical applications, oral poultices, and washings to heal disorders of the dermatological, digestive, gynecological, urinary, nervous, and respiratory systems [21].
8	*Iresine herbstii* Hook.	*Escancel*, *lancetilla*, *tigrecillo*	The isoflavanone 2′,2,5-trimethoxy-6,7-methylenedioxyisoflavanone, together with the isoflavone tlatlancuayin (2′,5-dimethoxy-6,7-methylenedioxyisoflavone) were isolated from the aerial parts [27].	Leaves, stalks, whole plant, stems, branches	Anti-inflammatory, anti-flu, analgesic, diuretic, sedative, and tonic. To treat intestinal, uterus, and vaginal infections, injuries, liver and kidney problems, general disorders of the gynecological, nervous, urinary, respiratory, dermatological, and digestive systems, mal aire (bad air) ^d^, and culture-related syndromes [4,16,20,21,22].
9	*Marchantia polymorpha* L.	*Sapo yuyu*	No information is reported in literature.	Whole plant	To heal body malaise [4].
	**APIACEAE**				
10	*Arracacia xanthorrhiza* Bancr. ^c^	*Zanahoria blanca*	This species is well known as food.	Leaves	To eliminate the cattle placenta [4].
11	*Eryngium foetidum* L.	*Culantro extranjero*	Phytochemical analysis of the leaves indicated the presence of flavonoids, tannins, a saponin and several triterpenoids, as well as the absence of alkaloids. A significant constituent of the EO of the plant is (*E*)-2-dodecenal (“eryngial”), accompanied by minor amounts of trimethylbenzaldehyde isomers. Pharmacological studies of the aerial parts have demonstrated anthelmintic activity due to eryngial, anti-inflammatory action due to the phytosterol fractions, anti-convulsant activity, and selective antibacterial activity against *Salmonella* and *Erwinia* species [28].	Whole plant	It is used to treat stomach pain [22].
	**APOCYNACEAE**				
12	*Lacmellea spaciosa* Woodson	*Chicle*	No information is reported in literature.	Fruits	Huaorani eat fruits. The latex from the trunk is used to clean teeth and as chewing gum [19].
13	*Marsdenia condurango* Rchb. f	*Condurango*	Pregnane glycosides isolated from the bark of *M. cundurango* were evaluated for their cytotoxic activity against human HL-60 leukemia cells, A549 lung adenocarcinoma cells, and TIG-3 normal lung cells. Moreover, a representative pregnane glycoside induced apoptosis in HL-60 cells [29].	Bark	It is used orally to treat general disorders of the digestive system [21].
	**ARECACEAE**				
14	*Ceroxylon parvifrons* (Engel) H. Wendl.	*Palma de ramos*	No information is reported in literature.	Leaves	The aerial parts are used as incense [4].
	**ASCLEPIADACEAE**				
15	*Orthosia ellemannii* (Morillo) Liede & Meve	*Cola de caballo*	No information is reported in literature.	Branches	It is used orally to treat general disorders of the urinary system [21].
	**ASTERACEAE**				
16	*Aequatorium jamesonii* (S.F. Blake) C. Jeffrey	*Guangalo*	No information is reported in literature.	Branches	Branches are rubbed to treat culture-related syndromes [21].
17	*Achyrocline hallii* Hieron	*Sacha algodón*, *lechugilla*	No information is reported in literature.	Leaves, whole plant	To treat disorders of the digestive system and injuries [4,21].
18	*Ambrosia arborescens* Mill.	*Marco*, *altamiso*	Sotillo et al. investigated the anticancer activity of sesquiterpene lactones isolated from the plant and a few synthetic derivatives against breast cancer cell lines, especially against cancer stem cells (CSCs) [30].	Leaves, branches	Pain relief (joints, head, throat), and to treat gastrointestinal, respiratory, and muscular problems. Topical applications and rubbings are also used to treat disorders of the dermatological system and culture-related syndromes [18,21].
19	*Ambrosia artemisioides* Meyen & Walpers ex Meyen	*Marco*	Compounds derived from allantolactone, as well as epieudesmane and oplopanone sesquiterpenes have been isolated from samples of *A. artemisioides* collected in the Tacna region of southern Peru [31].	Branches	To cure the fever or the cold caused by cold air or strong winds (locally known as mal aire in Spanish) ^d^ [4].
20	*Aristeguietia persicifolia* (Kunth) R.M. King & H. Rob	*Ishpingo*, *monte de culebra*	No information is reported in literature.	Branches	Branches are rubbed to treat culture-related syndromes [21].
21	*Artemisia sodiroi* Hieron	*Ajenjo*, *alcanfor*	A specimen collected in Ecuador gave a volatile fraction which contained sabinyl acetate (65.8%) as the main constituent [32].	Branches	Branches are rubbed to treat culture-related syndromes [21]. and gargles are used to heal disorders of the respiratory system [21].
22	*Baccharis oblongifolia* (Ruiz & Pav.) Pers.	*Chilca*	The flavonoids oblongifoliosides A and B have been isolated from the leaves [33].	Branches	To cure a restless and confused child, and in postpartum baths [4].
23	*Baccharis latifolia* (Ruiz & Pav.) Pers.	*Chilca larga*	A specimen collected in Ecuador afforded an essential oil, whose main components were limonene (33.72%), β-phellandrene (10.32%), sabinene (10.28%), β-pinene (6.99%), and α-pinene (5.44%). The essential oil exhibited moderate activity against *Trichophyton rubrum* (ATCC 28188) and *Trichophyton mentagrophytes* (ATCC 28185) [34].	Leaves, stalks	Pain relief (joints, head, throat) and to treat gastrointestinal, skin (inflammation, bruises), renal-urological, and neurological problems. Rubbings are used to treat culture-related syndromes [18,22].
24	*Bidens andicola* Kunth.	*Ñachic*, *nachag*	A new glycosyl chalcone ester, together with 7-*O*-glycosyl derivatives of flavonoids quercetin and quercetin 3-*O*-methyl ether have been isolated from the aerial parts. The sugar chains contained three or four sugar units, including β-D-glucopyranose, α-L-rhamnopyranose, and β-D-xylopyranose [35].	Whole plant, leaves	To decrease disease relapses after recovery (locally known as recaída in Spanish) and pain relief (joints, head, throat) [4,18].
25	*Bidens pilosa* L.	*Pacunga, amor seco*, *huichingue*	The isolation of sterols, terpenoids, phenylpropanoids and hydrocarbons were reported [36].	Whole plant, flowers	To decrease disease relapses after recovery (locally known as recaída), pain relief (at joints, head, throat), and as an anti-inflammatory [4,16].
26	*Bidens triplinervia* Kunth	*Ñachig*	No information is reported in literature.	Whole plant without roots	It is used orally to treat disorders of the gynecological system [21].
27	*Diplostephium oblanceolatum* S. F. Blake	*Chuquiragua*	No information is reported in literature.	Leaves	To heal body malaise [4].
28	*Gamochaeta americana* (Mill.) Wedd.	*Rabo de danta*, *lechuguilla*, *lancetilla*	No information is reported in literature.	Whole plant	To cure the cold [4].
29	*Loricaria thuyoides* (Lam.) Sch. Bip.	*Ushcu chaqui*, *pata de gallinazo*, *trensilla*	No information is reported in literature.	Branches	To cure a restless and confused child and used as a tonic and in energy baths [4].
30	*Oritrophium peruvianum* (Lam.) Cuatrec.	*Uña kushma*	No information is reported in literature.	Whole plant	To heal liver and kidney inflammations [4].
31	*Vernonanthura patens* (Kunth) H. Rob.	*Jujumba*	Lupeol was identified in the callus extract [37].	Leaves	It is used orally to treat disorders of the dermatological system [22].
	**BASELLACEAE**				
32	*Anredera ramosa* (Moq.) Eliasson.	*Lutuyuyu*	No information is reported in literature.	Whole plant	In baths for children, and to cure fever and headache [4].
	**BEGONIACEAE**				
33	*Begonia x tuberhybrida* Voss	*Begonia rosada*	No information is reported in literature.	Flowers, petals	To treat constipation [16]., and used as a sedative and tonic [20].
	**BETULACEAE**				
34	*Alnus acuminata* Kunth	*Aliso*	No information is reported in literature.	Leaves, buds	To cure headaches, and to treat bone fractures, sprains, and dislocations [4].
	**BRASSICACEAE**				
35	*Cardamine bonariensis* Pers.	*Berro*	No information is reported in literature.	Whole plant	It is used orally to cure disorders of the circulatory system [21].
36	*Lepidium chichicara* Desv.	*Chichira negra*	No information is reported in literature.	Whole plant	To decrease disease relapses after recovery (locally known as recaída in Spanish), to cure the fever or the cold caused by cold air or strong winds (locally known as mal aire (bad air) ^d^ [4].
37	*Lepidium thurberi* Wooton	*Chichira*	No information is reported in literature.	Plant without roots	It is used orally to treat gynecological disorders [21].
	**BROMELIACEAE**				
38	*Tillandsia straminea* Kunth	*Flor de cristo*, *clavel del aire*	No information is reported in literature.	Flowers	It is used to treat neurological disorders [21].
	**CACTACEAE**				
39	*Cumulopuntia corotilla* (K.Schum. ex Vaupel) E.F.Anderson	*Corotilla*	No information is reported in literature.	Whole plant	Pain relief (joints, head, throat), and to treat skin (inflammation, bruises) and neurological problems [18].
	**CAMPANULACEAE**				
40	*Siphocampylus scandens* (Kunth) G. Don	*Pena roja de monte*	No information is reported in literature.	Flowers	To treat neurological problems [4].
	**CANNACEAE**				
41	*Canna indica* L.	*Achira*	The phytochemical analysis showed the presence of alkaloids, carbohydrates, proteins, flavonoids, terpenoids, cardiac glycosides, oils, steroids, tannins, saponins, anthocyanin pigments, phlobatinins, and other chemical compounds. The pharmacological studies showed that this plant exerted antibacterial, antiviral, anthelmintic, molluscicidal, anti-inflammatory, analgesic immunomodulatory, antioxidant, cytotoxic, hemostatic, hepatoprotective, anti-diarrheal, and other effects [38].	Leaves	It is used to treat general neurological and respiratory problems [21,22].
42	*Canna coccinea* Mill.	*Platanillo*	No information is reported in literature.	Leaves, flowers	Pain relief (joints, head, throat) [18].
	**CAPPARACEAE**				
43	*Cleome longifolia* C. Presl.	*Sacha yuca*	No information is reported in literature.	Leaves	Antirheumatic [4].
	**CARICACEAE**				
44	*Carica pubescens* Lenn’e & C. Koch ^c^	*Chihualcán*, *chamburo*	Ethyl 3-*O*-β-D-glucopyranosyloxybutanoate, butyl 3-*O*-β-D-glucopyranosyloxybutanoate, and 3-oxo-octyl 1-*O*-β-D-glucopyranoside were isolated from fruit pulp by liquid chromatography on XAD [39].	Fruits, leaves	To cure nerves, diarrhea, and dislocations [4].
	**COMBRETACEAE**				
45	*Conocarpus erectus* L.	*Botoncillo*	The extracts of leaves, shoot, bark, and fruit showed high antibacterial, antioxidant, and hepta-protective activities due to phenolic content. Tannins and flavonoids were the main constituents. Tannins exhibited high antibacterial activity [40].	Flowers	Pain relief (joints, head, throat) [18].
	**COMMELINACEAE**				
46	*Callisia gracilis* (Kunth) R. D. Hunt	*Cachorillo*, *cachurillo*, *calcec*, *calcha verde*, *calsug*	No information is reported in literature.	Leaves	To cure general gynecological disorders [21,22].
47	*Callisia repens* (Jacq.) L.	*Calsi*, *calcha*, *calcec pequeño*	No information is reported in literature.	Leaves	To prevent postpartum relapse [4,21].
	**CLUSIACEAE**				
48	*Vismia baccifera* (L.) Triana & Planch.	*Achotillo*, *sangre de gallina*, *ushca*	Triprenylated anthranoids ferruginins A and B, together with ferruantrone and harunganin, were isolated from the taxon *V. baccifera* var. ferruginea [41].	Leaves	To treat skin conditions and fainting spells [19].
	**CUCURBITACEAE**				
49	*Cyclanthera pedata* (L.) Schrad. ^c^	*Achoccha*, *achogcha*, *caigua*	From a methanolic extract of the fruits flavonoid glycosides were separated by HPLC and identified [42].	Fruits	To cure earache and to decrease disease relapses after recovery (locally known as recaída in Spanish) [4].
	**EQUISETACEAE**				
50	*Equisetum bogotense* Kunth.	*Cola de caballo*, *caballo chupa*	No information is reported in literature.	Leaves, stalks, whole plant	Anti-inflammatory, antiseptic, depurative, diuretic, hepatic, febrifuge, anticancer, anticough, anti-parasite, and to cure kidney problems and liver inflammation [4,16,20,21].
51	*Equisetum giganteum* L.	*Chupa caballo*, cola de caballo	Caffeic acid derivatives, flavonoids, and styrilpyrones were identified. The most abundant glycosylated flavonoids were kaempferol derivatives [43].	Leaves, stalks	Pain relief (joints, head, throat), anti-inflammatory, and to treat gastrointestinal, respiratory, skin (inflammation, bruises), and renal-urological problems [9,21].
	**ERICACEAE**				
52	*Bejaria aestuans* L.	*Payana*, *payamo*, *payamo*	No information is reported in literature.	Flowers	To treat abdominal pain related to menstruation [4,21].
53	*Bejaria subsessilis* Benth.	*Pena de cerro*, *joyapa*	No information is reported in literature.	Flowers	To treat neurological problems [4].
54	*Cavendishia bracteata* (Ruiz & Pav. ex J. St.-Hil.) Hoerold	*Joyapa*, *salapa*	No information is reported in literature.	Fruits	Feed [4].
55	*Disterigma alaternoides* (Kunth) Nied.	*Perlillas o joyapilla*	No information is reported in literature.	Fruits	To treat physical exhaustion [4].
56	*Gaultheria erecta* Vent.	*Monte blanco*	No information is reported in literature.	Fruits	To treat physical exhaustion [4].
57	*Macleania rupestris* (Kunth) A. C. Sm.	*Joyapa*, *salapa verde*	No information is reported in literature.	Fruits	Antidiarrheal and to treat general physical malaise [4].
	**ERIOCAULACEAE**				
58	*Paepalanthus ensifolius* (Kunth) Kunth.	*Cucharillo*	No information is reported in literature.	Leaves	To cure nerves [4].
59	*Eriocaulon microcephalum* Kunth	*Monte de seguro*	No information is reported in literature.	Whole plant	To wish good luck [4].
	**EUPHORBIACEAE**				
60	*Cnidoscolus aconitifolius* (Mill.) I.M. Johnst	*Chaya*	Kaempferol, quercetin, and myricetin were the most abundant phenolic compounds found in an extract [44].	Leaves	To treat general digestive and circulatory problems [21].
61	*Sapium glandulosum* (L.) Morong	*Caucho*	LC-MS analysis of the latex revealed the presence of tigliane-type diterpenoids, especially 12-deoxyphorbol esters. Considering that 12-deoxytigliane diterpenes are described as antitumor and antiviral agents, these results indicated that this plant has pharmacological potential [45].	Leaves	An infusion of burnt leaves is used to remove pimples from the skin. The leaves are used to cure fainting [19].
	**FABACEAE**				
62	*Acacia macracantha* Humb. & Bonpl. ex Willd.	*Uña de gato*	The sugars identified in gum exudates of eight specimens of *A. macracantha* collected in Venezuela were galactose, arabinose, glucuronic acid, 4-*O*-methylglucuronic acid, and rhamnose [46].	Leaves, flowers	Pain relief (joints, head, throat), anti-inflammatory, and to treat gastrointestinal, skin (inflammation, bruises), and renal-urological problems [18].
63	*Amicia glandulosa* Kunth	*Nona*, *urusus*, *orozús*	No information is reported in literature.	Flowers	To treat respiratory disorders [21].
64	*Desmodium molliculum* (Kunth) DC.	*San Antonio, hierba de san Antonio*, *hierba del ángel*	No information is reported in literature.	Plant without roots	To treat gynecological disorders [21].
65	*Myroxylon balsamum* (L) Harms	*Chaquino*	(±)-7-Hydroxy-4′-methoxyisoflavanone, (±)-7,3′-dihydroxy-4′-methoxyisoflavanone, and 2-(2′,4′-dihydroxyphenyl)-5,6-dimethoxybenzofuran were isolated from this species [47].	Bark	To treat digestive disorders [21].
66	*Myroxylon peruiferum* L. f.	*Chaquino*	Two flavonoids, 2′-hydroxy-7,3′,4′-trimethoxyisoflavanone, and 2′-hydroxy-7,3′,4′-trimethoxyisoflavone were isolated from this species [48].	Bark	To treat general respiratory disorders [22].
	**GENTIANACEAE**				
67	*Halenia weddelliana* Gilg	*Taruka cacho*, *cacho de venado*	No information is reported in literature.	Whole plant	It helps maintain milk production in cattle [4].
68	*Macrocarpaea lenae* J. R. Grant	*Tabaco de cerro*	No information is reported in literature.	Leaves	To cure the fever or the cold caused by cold air or strong winds (locally known as mal aire (bad air) ^d^ [4].
	**GERANIACEAE**				
69	*Geranium diffusum* Kunth.	*Cáncer*	No information is reported in literature.	Whole plant	To cure gangrene and infections after birth [4].
	**JUGLANDACEAE**				
70	*Juglans neotropica* Diels. ^c^	*Nogal*, *tocte*	No information is reported in literature.	Leaves	In postpartum baths, and to treat disorders of the circulatory system [4,21,22].
	**LAMIACEAE**				
71	*Hyptis purdiei* Benth.	*Poleo de cerro*, *poleo negro*	No information is reported in literature.	Branches	The plant is rubbed to treat culture-related syndromes [21].
72	*Minthostachys mollis* (Kunth) Griseb.	*Poleo blanco*, *tipo*	This aromatic shrub grows wild in the Andes above 1500 m of altitude from Venezuela to Argentina. Apparently, the composition of the essential oil of the plant grown in different geographical locations is not the same. A specimen from Argentina contained (-)-menthone as the main component; the oil from *M. mollis* collected in Ecuador contained neomenthol, (-)-menthone and menthol as the main constituents, while pulegone (75.2–79.3%) predominated among 28 components identified in the oil from Venezuela [49].	Branches	To cure the fever or the cold caused by cold air or strong winds (locally known as mal aire (bad air) ^d^, for pain relief (joints, head, throat), anti-inflammatory, and to cure respiratory problems [4,18,21].
73	*Salvia leucocephala* Kunth.	*Espliego*, *lavanda*	No information is reported in literature.	Whole plant	In postpartum baths [4].
74	*Salvia scutellarioides* Kunth.(syn. *S. palaefolia*)	*Matico grande*, *salvia flor azul*	Alkaloids, triterpenes, and lignans were isolated this species [50].	Flowers	Vaho de agua (supernatural disease caused by exposure to water-vapors from rivers, lakes, etc.) ^d^ [4,21].
	**LAURACEAE**				
75	*Persea americana* Mill.	*Aguacate*, *palta*	Juglanin and (+)-lyoniresinol were isolated from the leaves. Both compounds showed significant cell regeneration in neomycin-damaged hair cell without cellular toxicity [51].	Seeds	To treat coups and hematomas [4].
	**LYCOPODIACEAE**				
76	*Huperzia sellifolia* B. Øllg.	*Wuaminga colorado*	No information is reported in literature.	Whole plant	Amulet against evil eye and sorcery ^d^ [4].
77	*Lycopodium weberbaueri* (Nessel).	*Wuaminga suco or gris*	No information is reported in literature.	Whole plant	Amulet against evil eye and sorcery ^d^ [4].
78	*Huperzia austroecuadorica* B. Øllg	*Wuaminga verde* (*pequeña*)	No information is reported in literature.	Whole plant	Amulet against evil eye and sorcery ^d^ [4].
	**MELASTOMATACEAE**				
79	*Aciotis rubricaulis* (Mart. ex DC.) Triana	*Chulco*	No information is reported in literature.	Leaves, stalks	Pain relief (joints, head, throat), and to treat gastrointestinal and renal-urological problems [18].
80	*Brachyotum confertum* (Bonpl.) Triana.	*Sacha zarcillo*	No information is reported in literature.	Branches	Against allergies [4].
81	*Tibouchina laxa* (Desr.) Cogn.	*Dumaricgri*, *dumarín*, *chininingue*, *garra del diablo*	No information is reported in literature.	Flowers	To treat eye infections of guinea pigs (it has not been used for man) [4].
	**MELIACEAE**				
82	*Cedrela montana* Moritz ex Turcz.	*Cedro andino*, *cedro blanco*	Two oleanane-type triterpenes, 3-oxo-11a,12a-epoxy-oleanan-28,13b-olide and 3-oxo-olean-11-en-28,13b-olide, were isolated from the fruits and seeds. In addition, the known compounds oleanonic acid, a mixture of b-sitosterol and stigmasterol, and the limonoid photogedunin were isolated [52].	Leaves	In postpartum baths and to relieve bone pain [4].
	**MORACEAE**				
83	*Ficus yoponensis* Desv.	*Saumerio*	No information is reported in literature.	Latex	The latex has medicinal and technological uses and serves as an adhesive to bandage wounds. To treat kidney diseases and rheumatic pain, stomach pain and ulcers, varicose veins, hepatic inflammatory processes, and used as a vermifuge [19].
	**MYRICACEAE**				
84	*Morella parvifolia* (Benth.) C.Parra	*Laurel*, *laurel de cera*, *laurel de monte*	The main constituents of the essential oil analyzed by GC-MS were α-bisabolol (50.6–58.9%) and α-pinene (12.9–16.8%). No antibacterial activity was detected [53].	Branches	To treat general gynecological disorders [21].
85	*Morella pubescens* (Humb. & Bonpl. Ex Willd.) Wilbur	*Laurel*, *laurel de cera*	No information is reported in literature.	Branches	To treat general gynecological disorders [21].
86	*Myrica parvifolia* Benth.	*Laurel*	No information is reported in literature.	Branches, buds	To treat the fever or the cold caused by cold air or strong winds (locally known as mal aire (bad air) ^d^, used against stomach colic, and to treat fainting during childbirth [4].
87	*Myrica pubescens* Humb. & Bonpl. ex Willd.	*Millma laure* (*laurel lanudo*)	No information is reported in literature.	Leaves	To treat the fever or the cold caused by cold air or strong winds (locally known as mal aire (bad air) ^d^ [4].
	**MYRTACEAE**				
88	*Psidium guajava* L.	*Guayaba*	Meroterpenoids, a triterpenoid, terpenoid derivatives, and aromatic compounds, were isolated from the leaves. Meroterpenoids were evaluated for their antitumor and antifungal activities. Meroterpenoids psiguajadial D, guapsidial A, 4,5-diepipsidial A, guadial A, and guadial B were cytotoxic against five human tumor cell lines (HL-60, A-549, SMMC-7721, MCF-7, and SW-480). Guapsidial A was the most effective with an IC_50_ of 3.21–9.94 μmol·L^−1^ [54].	Fruits	It is used orally to treat disorders of the digestive system [21].
	**ONAGRACEAE**				
89	*Fuchsia harlingii* Munz	*Pena*, *pena de cerro*	No information is reported in literature.	Flowers	Anti-inflammatory and sedative remedy [20]. It is used orally to treat neurological disorders [21].
90	*Fuchsia hypoleuca* I. M. Johnst	*Sacha pena*	No information is reported in literature.	Flowers	To treat neurological disorders [4].
91	*Fuchsia loxensis* Kunth	*Pena*, *pena rosada*	No information is reported in literature.	Flowers	Cardiotonic, febrifuge and sedative remedy [20]. It is used orally to treat neurological disorders [21].
92	*Ludwigia nervosa* (Poir.) H. Hara	*Flor de reina*, *mejorana de huerta*	No information is reported in literature.	Flowers	Anti-inflammatory and sedative remedy [20]. It is used orally to treat gynecological, nervous, and dermatological disorders [21].
93	*Oenothera rosea* L’Her. ex Aiton	*Shullo*, *shullu colorado*	The flavonoids: quercetrin and quercetin 3-*O*-β-D-allopyraNoside-3″,6″-diacetate were isolated from this plant [55].	Flowers, leaves, stalks	Anti-inflammatory, digestive, diuretic remedy, and to treat hepatic and kidney problems [16,21,22].
	**ORCHIDACEAE**				
94	*Epidendrum cochlidium* Lindl.	*Flor de cristo anaranjada*	No information is reported in literature.	Flowers	To treat neurological disorders [4].
95	*Epidendrum fimbriatum* Kunth	*Flor de cristo blanca*, *espíritu*	No information is reported in literature.	Flowers	To treat internal tumors [4].
96	*Epidendrum jamiesonis* Rchb.f.	*Flor de cristo violeta*, *maywa*	No information is reported in literature.	Flowers	Used as an anti-inflammatory, sedative, diuretic, and hepatic remedy [20]. It is used orally to treat dermatological disorders [21].
	**OXALIDACEAE**				
97	*Oxalis corniculata* L.	*Chulco*, *trebol*, *trebol de huerta*	Corniculatin A was isolated from an EtOAc extract of the whole plant, together with luteolin, luteolin-7-*O*-β-D-glucoside, and β-sitosterol-3-*O*-β-D-glucoside [56].	Whole plant	Against scurvy (scorbutic tongue) [4].
98	*Oxalis peduncularis* Kunth.	*Chulco amarillo*	No information is reported in literature.	Whole plant	To cure infection of the throat [4].
99	*Oxalis spiralis* Ruiz & Pav. ex G. Don	*Chulco*, *cañitas*, *trigonella*	No information is reported in literature.	Whole plant	To cure infection of the throat [4].
	**PHYTOLACCACEAE**				
100	*Phytolacca americana* L.	*Atuczara*, *hatun sara*	No information is reported in literature.	Fruits	Against dandruff [4].
	**PIPERACEAE**				
101	*Peperomia blanda* (Jacq.) Kunth	*Sacha congona*	Tetrahydrofuran lignans and flavones were isolated from the aerial parts. Some lignans exhibited high in vitro trypanocidal activity against epimastigotes of *Trypanosoma cruzi* strain Y. [57].	Plant roots	It is used orally to treat neurological disorders [21].
102	*Peperomia congona* Sodiro	*Congona*, *congona olorosa*	No information is reported in literature.	Leaves, flowers, stalks	Anti-parasitic, antiperspirant, analgesic, cardiotonic, diuretic, hepatic, sedative, and to treat headache and insomnia [4].
103	*Peperomia galioides* Kunth	*Tigresillo*, *sacha congona*, *congona de cerro*	Eighty-four constituents were identified in the leaf essential oil analyzed by GC and GC-MS, which constituted more than 99% of the oil. The main components were safrole (42.3%) and *epi*-α-bisabolol (29.2%) [58].	Whole plant	Against aire de agua o vaho de agua (a supernatural disease caused by exposure to water-vapors from, for example, rivers, lakes, etc.) ^d^ [4,21].
104	*Peperomia ilaloensis* Sodiro	*Congona de castilla*, *congona negra*	No information is reported in literature.	Plant without roots	As an analgesic and sedative remedy [20]. It is used orally to treat neurological and sensorial disorders [21].
105	*Peperomia peltigera* C. DC. ^c^	*Pata conguyo*, *condorcol*	No information is reported in literature.	Fruit, leaves	To treat headache, respiratory, and neurological problems [4,18].
106	*Piper aduncum* L.	*Cordoncillo*, *matico de monte*, *monte del soldado*	Bioactivity-guided fractionation of a leaf ethanolic extract afforded the dihydrochalcone adunchalcone, which was evaluated against promastigote forms of *Leishmania amazonensis*, *L. braziliensis*, *L. shawi*, and *L. chagasi*. The compound displayed EC_50_ values of 11.03, 26.70, and 11.26 μM, respectively, as well as selective indexes of 4.86, 2.01, 4.76, and 0.50, respectively. In contrast, adunchalcone exhibited weak activity against intracellular forms of *L. amazonensis*, compared to amphotericin B [59].	Leaves, stalks	To treat infections of external wounds, gastrointestinal, respiratory, and skin (inflammation, bruises) problems, and an anti-inflammatory [4,18,21,22].
107	*Piper crassinervium* Kunth	*Guabiduca dulce*	Bioactivity-guided fractionation of a leaf extract afforded three antifungal prenylated hydroquinones, together with two antifungal flavanones [60].	Leaves	As an analgesic and antiseptic remedy, and against stomachache [20]. It is used orally to treat hormonal and respiratory disorders [21].
	**POLYGALACEAE**				
108	*Polygala paniculata* L.	*Mentol, poligaga flores violetas*	The xanthones 1-hydroxy-5-methoxy-2,3-methylenedioxy-xanthone and 1,5-dihydroxy-2,3-dimethoxyxanthone, together with the coumarin murragatin and the flavonol rutin were isolated from this plant [61].	Whole plant	It is used orally to treat musculoskeletal disorders [21].
	**POLYGONACEAE**				
109	*Rumex tolimensis* Wedd	*Turu*	No information is reported in literature.	Stems, leaves	To promote hair growth and against dandruff [4].
	**POLYPODIACEAE**				
110	*Niphidium crassifolium* (L) Lellinger	*Calaguala*, *calawala*	No information is reported in literature.	Roots	It is used orally to treat digestive and urological disorders [21].
	**PTERIADACEAE**				
111	*Adiantum poiretii* Wikstr.	*Culantrillo pata negra*	No information is reported in literature.	Whole plant, leaves	To treat the cold [4]. It is used orally to treat gynecological disorders [21].
112	*Adiantum raddianum* C. Presl.	*Culantrillo*	No information is reported in literature.	Leaves	It is used orally to treat gynecological and urological disorders [21].
113	*Cheilanthes bonariensis* (*Willd*.) Proctor.	*Helecho congona*	No information is reported in literature.	Leaves	It is used orally to treat gynecological disorders [21].
114	*Notholaena sulphurea* (Cav.) J. Sm.	*Grano de oro*	The main constituent of the yellow frond exudate of this fern was identified as 3,5,2′-trihydroxy-7-methoxy-8-acetoxy flavone. The 5,2′-dihydroxy-7,8-dimethoxy flavone was also found, along with some common flavonoids. The white form of the fern produced three dihydrochalcones that were accompanied by some kaempferol methyl ethers and apigenin-7-methyl ether. The 3-acetoxy as well as the 3-butyryloxy and the 4′-butyryloxy derivatives of 7-methyl aromadendrin were also identified [62].	Leaves	It is used orally to treat gynecological disorders [21].
115	*Pityrogramma ebenea* (L) Proctor.	*Doradilla plateada*, *luna plateada*	2′,6′-Dihydroxy-4,3′-dimethoxy-4′,5′-dioxymethylenedihydrochalcone was identified in the leaves [63].	Leaves	It is used orally to treat gynecological disorders [21].
116	*Pityrogramma calomelanos* (L.) Link	*Doradilla del sol*	An isolated new pigment was assigned the structure of an 8- or 6-dihydrocinnamoyl-5,7-dihydroxy-4-phenyl-2H-1-benzopyran-2-one. From the same fern, two other phenyl-benzopyran-2-one-derivatives and a 2-phenyl-γ-pyron (ol)-ring derivative were isolated [64].	Leaves	It is used orally to treat gynecological disorders [21].
117	*Trachypteris induta* (Maxon) R.M. Tryon & A.F. Tryon	*Pata de gallina*	No information is reported in literature.	Leaves	It is used orally to treat gynecological disorders [21].
	**RANUNCULACEAE**				
118	*Clematis haenkeana* C. Presl.	*Zarzaparrilla roja*	No information is reported in literature.	Buds	To cure sore teeth [4].
	**ROSACEAE**				
119	*Alchemilla aphanoides* Mutis ex L f.	*Saucillo*	No information is reported in literature.	Branches	It is used orally to treat neurological disorders [21].
120	*Hesperomeles obtusifolia* (Pers.) Lindl	*Quique*, *cerote*	No information is reported in literature.	Leaves	Pain relief (joints, head, throat), and to cure gastrointestinal, respiratory, and renal-urological disorders [18].
121	*Margyricarpus pinnatus* (Lam.) Kuntze	*Perlilla*, *nigua*	The main constituents of the leaf essential oil were limonene (57.8%) and α-pinene (9.7%), whereas sabinene (24.2%), limonene (9.1%), and pinocarvone (9.7%) were the main components of the fruit oil [65].	Plant without roots	It is used orally to treat respiratory and dermatological disorders [21].
122	*Prunus serotina* Ehrh.	*Capulí*	Bio-guided fractionation of a methanolic extract afforded 2,3-dihydro-5,7-dihydroxy-2-(4-hydroxyphenyl)-4H-1-benzopyran-4-one (naringenin, NGN), 3,4,5-trimethoxybenzoic acid, and 1,3,5-trimethoxybenzene. NGN exhibited in vitro activity, in a time-concentration-dependent manner (EC_50_ = 89.3 μM]. Furthermore, NGN at a dose of 376.1 μmol/kg, displayed in vivo efficacy against *Taenia crassiceps* cysts similar to albendazole at 188.4 μmol/kg [66].	Leaves	In postpartum baths and to cure bone pain [4].
123	*Rubus urticifolius* Poir	*Mora silvestre*	No information is reported in literature.	Bud and flowers	To cure gangrene [4].
	**RUBIACEAE**				
124	*Cinchona pubescens* Vahl	*Cascarilla*, *cascarilla roja*	Seven known anthraquinones, alizarin-2-methylether, anthragallol-1,2-dimethylether, purpurin, purpurin-1-methylether, 1-hydroxy-2-hydroxymethylanthraquinone, 2-hydroxy-1,3,4-trimethoxyanthraquinone, and 2,5-(or 3,5-)dihydroxy-1,3,4-(or 1,2,4-)trimethoxyanthraquinone, together with five new anthraquinones, 2-hydroxy-1,3,4,6-(or 1,3,4,7-)tetramethoxyanthraquinone, 1,6-(or 1,7-)dihydroxy-2-methylanthraquinone, 5-hydroxypurpurin-1-methyl ether, 4,6-(or 4,7-)dihydroxy-2,7-(or 2,6-)dimethoxyanthraquinone, and 6,7-dihydroxy-1-methoxy-2-methylanthraquinone were isolated from callus cultures [67].	Bark	It is used orally to treat respiratory problems [21,22].
	**SAPOTACEAE**				
125	*Pouteria caimito* (Ruiz & Pav.) radlk	*Caimito*	Three triterpenoids, Δ^14^–taraxene–3β–ol acetate, Δ^14^–taraxene–3–one, and Δ^14^–taraxene–3β–ol, together with β–sitosterol, were isolated from the bark [68].	Leaves	The latex is used to remove subcutaneous larvae. The leaf infusion is used to treat skin infections [19].
	**SCROPHULARIACEAE**				
126	*Pedicularis incurva* Benth.	*Pimpinela del cerro*	No information is reported in literature.	Branches	To treat the cold [4].
	**SOLANACEAE**				
127	*Brugmansia candida* Pers.	*Floripondio blanco*, *guando*, *guando blanco*	The alkaloids, scopolamine and anisodamine, were produced in a modified bioreactor culture system [69].	Flowers, leaves	To cure the fever or the cold caused by cold air or strong winds (locally known as mal aire (bad air) ^d^ [4,21,22].
128	*Brugmansia sanguinea* (Ruiz & Pav.) D. Don	*Floripòndio rojo*, *guando rojo*	No information is reported in literature.	Flowers, leaves	The plant is rubbed to treat culture-related syndromes [21].
129	*Cestrum mariquitense* Kunth	*Sauco negro*	No information is reported in literature.	Branches	It is used to treat general disorders of the circulatory system [21].
130	*Cestrum racemosum* Ruiz & Pav	*Sauco blanco*	No information is reported in literature.	Branches	It is used to treat general culture-related syndromes [21,22].
131	*Cestrum sendtnerianum* C. Mart.	*Sauco negro*	No information is reported in literature.	Leaves and flowers	To cure fever, headache, and postpartum relapses [4].
132	*Cyphomandra betacea* (Cav.)	*Tomate de árbol*	No information is reported in literature.	Fruits	To cure throat infection [4].
133	*Lycopersicon hirsutum* Dunal	*Monte de guishco*, *monte de gallinazo*, *monte de ushco*	No information is reported in literature.	Branches, leaves	It is used to treat general culture-related syndromes [22].
134	*Physalis peruviana* L. ^c^	*Uvilla*, *uchuva*, *uvilla lanuda*	The UPLC-ESI-MS/MS metabolic profile of an EtOAc extract of fruits cultivated in Egypt allowed the identification of several phenolic compounds. Moreover, the EtOAc extract showed remarkable α-amylase, β-glucosidase, and lipase inhibitory effects. In an in vivo antihyperglycemic test with streptozotocin (STZ)-induced diabetic rats, the EtOAc extract decreased the blood glucose level, prevented the reduction of body weight, and improved serum indicators of kidney injury [70].	Fruits	To lower cholesterol [4].
135	*Solanum americanum* Mill.	*Mortiño*, *hierba mora*	No information is reported in literature.	Leaves, fruits	Anti-inflammatory, analgesic, digestive, febrifuge, sedative, to treat respiratory diseases, the fever, the cold, pneumonia, internal infections, and kidney problems [4,16,21,22].
136	*Solanum juglandifolium* Dunal	*Matico*	No information is reported in literature.	Flowers	Against air wáter ^d^ [4].
137	*Solanum nigrescens* M.	*Hierba mora*	The antifungal activity of the extracts was attributed to the presence of a spirostanol glycoside, cantalasaponin-3 [71].	Leaves	Pain relief (joints, head, throat), and to cure fever, gastrointestinal, respiratory, skin (inflammation, bruises), renal-urological, and anti-inflammatory diseases [4,21].
138	*Solanum oblongifolium* Dunal, Solan.	*Turpe*, *tululuche*, *mata perro*	No information is reported in literature.	Branches, leaves	To cure the fever or the cold caused by cold air or strong winds (locally known as mal aire (bad air) ^d^, and dislocation [4].
139	*Solanum pimpinellifolium* L.	*Monte de gallinazo*	No information is reported in literature.	Branches	To treat general culture-related syndromes [21].
	**TILIACEAE**				
140	*Triumfetta althaeoides* Lam.	*Abrojo*, *achotillo*, *cadillo*	No information is reported in literature.	Leaves	To treat liver and kidney inflammations [4,22].
141	*Triumfetta semitriloba* Jacq.	*Abrojo*, *cadillo*, *monstrante*	No information is reported in literature.	Leaves	To treat general urological diseases [21].
	**VALERIANACEAE**				
142	*Valeriana pyramidalis* Kunth	*Valeriana*	No information is reported in literature.	Roots	It is used orally to treat neurological problems [21].
143	*Valeriana microphylla* Kunth	*Valeriana de cerro*	Five valepotriates, i.e., valtrate, isovaltrate, diavaltrate, acevaltrate, and didrovaltrate, together with nardostaquine, and two lignans, (+)-1-hydroxypiNoresiNol and pinoresinol were isolated and identified [72].	Roots	To cure nerves [4].
	**VERBENACEAE**				
144	*Aloysia citriodora* Paláu	*Cedrón*	The effect of continuous and pulsed ultrasound pre-treatments (15, 30, and 45 min), followed by conventional hydrodistillation, on the characteristics of isolated essential oils (EOs) from dried leaves of *A. citriodora* was evaluated for the first time. Moreover, the chemical composition, the antibacterial and antioxidant activities, as well as the contents in heavy metals (iron, copper, lead, arsenic, and cadmium) of the Eos were determined [73].	Leaves	Pain relief (joints, head, throat), anti-inflammatory, and to treat gastrointestinal and respiratory problems [4].
145	*Aloysia triphylla* (L’Hér.) Britton. *	*Cedrón*	The EO contained myrcenone (36.50%), α-thujone (13.10%), lippifoli-1(6)-en-5-one (8.87%), and limonene (6.87%) as the main components [74].	Leaves, flowers, stalks	Anti-inflammatory, antispasmodic, anti-neuralgic, analgesic, cardiotonic, digestive, stomach tonic, diuretic, and to cure the fever, headache, the cold, and colic [4,21,22].
146	*Phyla strigulosa* (M. Martens & Galeotti) Moldenke	*Sistalgina/novalgina*	No information is reported in literature.	Whole plant	To treat stomachache [22].
147	*Verbena litoralis* Kunth	*Verbena*	Phytochemical tests revealed the presence of iridoid glycosides, flavonoids, phenylpropanoid derivatives, phenylethanoid derivatives, cinnamic acid derivatives, and triterpenes. The extract was classified ‘safe’ (category 5), according to the OECD guidelines, in acute treatments [75].	Flowers	To cure plagues and headache, body malaise, infection of the throat, respiratory and skin diseases (inflammation, bruises), flu, and pain relief (joints, head, throat) [4,18,21,22].
	**VIOLACEAE**				
148	*Viola arguta* Willd. & Schult. ex Roem.	*Violeta de campo flor roja*, *pucango*	No information is reported in literature.	Flowers	To cure nerve problems [4].
149	*Viola dombeyana* DC.	*Violeta de campo*	No information is reported in literature.	Flowers	To cure nerve problems [4].
	**VISCACEAE**				
150	*Dendrophthora fastigiata* Kuijt.	*Suelda pequeña*	No information is reported in literature.	Whole plant	To treat fractured and dislocated bones [4].
151	*Phoradendron parietarioides* Trel.	*Suelda grande*, *matapalo*, *solda-solda*, *suelda*	No information is reported in literature.	Whole plant	To treat fractured and dislocated bones [4].
	**WINTERACEAE**				
152	*Drimys granadensis* L. f.	*Cascarilla*	A total of 85 components were identified in the leaf EO analyzed by GC and GC-MS. Germacrene D (14.7%), sclarene (9.5%), α-cadinol (7.3%), longiborneol acetate (6.3%), drimenol (4.2%), (*Z*)-β-ocimene (3, 4.2%), α-pinene (3.2%), and β-elemene (2.7%) were the main components of the oil. The EO was also tested against eight bacteria strains using the Kirby–Bauer disk-diffusion method. Most of the tested Gram-positive bacteria were susceptible to the oil, while the Gram-negative bacteria were not [76].	Bark	To cure sore teeth [4].

^a^ The names of endemic plants have been underlined. ^b^ Data obtained by research groups working in countries other than Ecuador. ^c^ The plant is also cultivated. ^d^ A supernatural disease.

**Table 2:** Botanical and vernacular names, used part(s), and traditional uses of introduced medicinal plants.

No.	Botanical Name	Vernacular Name	Used Part(s) of the Plant	Traditional Uses
	**AGAVACEAE**			
1	*Agave americana* L. ^a,b^	*Cabuya*, *penco*, *chaguarquero*	Stems	To heal bone fractures and dislocations [4].
	**AIZOACEAE**			
2	*Mesembryanthemum elegans* L.	*Condorcoles pequeño*	Leaves	To treat nerves and headache [4].
	**AMARANTHACEAE**			
3	*Aerva sanguinolenta* (L.) Blume ^a,b^	*Escancel*	Whole plant without roots	It is used in topical applications, orally, and in poultices and washings to treat general disorders of the dermatological, digestive, gynecological, urinary, and nervous systems, and to cure renal problems and culture-related syndromes [18,21].
4	*Dysphania ambrosioides* (L) Mosyakin & Clemants ^a,b^	*Paico*	Branches	Branches are rubbed to treat disorders of the digestive system and culture-related syndromes [21,22].
	**APIACEAE**			
5	*Anethum graveolens* L. ^a,b^	*Eneldo*	Whole plant	Pain relief (joints, head, throat), and to treat gastrointestinal, respiratory, skin (inflammation, bruises), and renal-urological problems [18].
6	*Apium graveolens* L. ^a,b^	*Apio*	Leaves, stalks	Pain relief (joints, head, throat), and to treat gastrointestinal, respiratory, and anti-inflammatory problems [18].
7	*Apium leptophyllum* (Pers.) F. Muell. ^b^	*Culantrillo blanco*	Whole plant	It is used to treat the cold [4].
8	*Coriandrum sativum* L. ^a,b^	*Cilantro*, *culantro*	Whole plant	To treat the abdominal pain related to menstruation [4].
9	*Cyclospermum leptophyllum* (Pers.) Sprague ex Britton & P. Wilson ^b^	*Culantrillo*, *cominillo*	Branches	It is used orally to treat disorders of the digestive system [21].
10	*Daucus carota* L. ^a,b^	*Zanahoria*	Leaves	The juice is used to treat gastritis [4].
11	*Foeniculum vulgare* Mill. ^a,b^	*Hinojo*, *eneldo*	Leaves, whole plant	It is used orally to treat disorders of the digestive system, and as an anti-inflammatory, a relaxant, against conjunctivitis, indigestion, gastritis, menstrual colic, diabetes, anticancer, and to increase the breast milk [4,16,21].
12	*Petroselinum crispum* (Mill.) Fuss ^a,b^	*Perejil*	Whole plant, leaves, stalks	Pain relief (joints, head, throat), and to treat gastrointestinal, respiratory, and neurological disorders [4,18].
13	*Pimpinella anisum* L. ^b^	*Anís*	Seeds	Pain relief (joints, head, throat), to treat gastrointestinal problems, and as a febrifuge [18].
	**ASPHODELACEAE**			
14	*Aloe vera* (L.) Burm. f. ^b^	*Sábila*	Leaves	Pain relief (joints, head, throat), and to treat gastrointestinal, respiratory, and renal-urological problems. Topical applications are used to treat skin problems [18,21,22].
	**ASTERACEAE**			
15	*Ageratum conyzoides* L. ^b^	*Canayuyo*, *pedorrera, hierba de chivo*	Whole plant	To heal gangrene and infections. It is also used orally to treat disorders of the digestive system [4,21].
16	*Cotula australis* (Sieber ex Spreng.) Hook.f.	*Chichira sombrerito*	Whole plant	To decrease disease relapses after recovery (locally known as recaída in Spanish) [4].
17	*Cynara cardunculus* L. ^a,b^	*Alcachofa*	Fruits	It is used orally to treat disorders of the hormonal system [21].
18	*Matricaria chamomilla* L. ^a,b^	*Manzanilla*	Whole plant	Pain relief (joints, head, throat), anti-inflammatory, to treat gastritis, gastrointestinal and respiratory problems, skin inflammation, and bruises [4,18,22].
19	*Matricaria recutita* L. ^a,b^	*Manzanilla*	Flowers, leaves, stalks	Anti-inflammatory, sedative, anti-flatulence, anthelmintic, analgesic, carminative, digestive, febrifuge, and used against cramps, insomnia, wounds, stomach pain, and burns. Used as a stimulant tonic. It is also used in gargles to treat disorders of the respiratory system [16,21].
20	*Sigesbeckia mandoni* Schult. Bip.	*Sacha jícama*	Leaves	To treat diarrhea in children from 1 to 6 months of age [4].
21	*Sonchus oleraceus* L. ^a,b^	*Cerraja, serraja*, *Cachicerraja*	Whole plant	To heal body malaise, pain relief (joints, head, throat), and to treat gastrointestinal, respiratory, and renal-urological problems, skin inflammation, and bruises [4,18].
22	*Tagetes erecta* L. ^a,b^	*Killo rosa*, *flor de muerto*, *calendula*	Branches, flowers	Against vaho de agua ^c^ (a supernatural disease, presumed to be due to exposure to water-vapors). The plant is rubbed to heal culture-related syndromes [4,21].
23	*Tagetes patula* L. ^a,b^	*Arrayosa*	Flowers	The plant is rubbed to heal culture-related syndromes [22].
24	*Tanacetum parthenium* (L.) Sch. Bip. ^b^	*Santa María*	Whole plant	To cure fear in children [4,21,22].
25	*Taraxacum officinale* F. H. Wigg. ^b^	*Diente de león*, *taraxaco*	Whole plant	To cure gastritis and ulcer, and for pain relief (joints, head, throat). To treat gastrointestinal, respiratory, and renal-urological problems, skin inflammation, and bruises [4,18,21].
	**BALSAMINACEAE**			
26	*Impatiens balsamina* L. ^a,b^	*Amor constante*, *begonia*	Flowers	In postpartum relapse [4].
	**BORAGINACEAE**			
27	*Borago officinalis* L. ^a,b^	*Borraja*	Flowers, leaves, stalks	Anti-inflammatory, anti-flu, expectorant, febrifuge, to enhance blood circulation, sudorific, astringent, diuretic, anti-hypercholesterolemic, analgesic, antidiarrheal, antitussive and emmenagogue; to treat hepatic pain, conjunctivitis, burnings, headache, and coughs; to decrease disease relapses after recovery (locally known as recaída in Spanish); to cure gastrointestinal, respiratory, and renal-urological problems [16,18,20,21,22].
28	*Symphytum officinale* L. ^b^	*Consuelda*, *suelda*	Leaves	It is used to treat musculoskeletal disorders [21].
	**BRASSICACEAE**			
29	*Brassica oleracea* ‘Acephala’ ^a,b^	*Col silvestre*	Stems	To cure liver and kidney inflammations and infections, and postpartum infections [4].
30	*Matthiola incana* (L.) R. Br. ^a,b^	*Alhelí*, *alelí*	Flowers	It is used orally to treat neurological disorders [21].
31	*Nasturtium officinale* R. Br. ^a,b^	*Berro chico*, *berro negro*	Leaves, whole plant	To cure body malaise, headache, flu, and pneumonia [4].
32	*Rorippa nasturtium-aquaticum* (L.) Hayek	*Berro*	Plant without roots	It is used orally to cure disorders of the circulatory system [21].
	**CACTACEAE**			
33	*Echinopsis pachanoi* (Britton & Rose) Friedrich & G. D. Rowley	*San pedrillo*, *san Pedro, aguacolla*	Stems	In sorcery rituals ^c^ [4,22].
34	*Trichocereus macrogonus* (Salm-Dyck) Riccob.	*San pedrillo*	Wood	To treat culture-related syndromes [21].
	**CAMPANULACEAE**			
35	*Lobelia* cf. *decurrens* Cav. ^a^	*Cholo valiente*, *cararango*	Branches	To treat culture-related syndromes [21,22].
	**CAPRIFOLIACEAE**			
36	*Sambucus nigra* L. ^a,b^	*Tilo*, *sauco tilo*	Flowers	Anti-flu, to treat bronchitis, febrifuge, antidiarrheal, sedative, antitussive, to cure nerves, colds, coughs, and headaches [16,20,21,22].
	**CARYOPHYLLACEAE**			
37	*Dianthus caryophyllus* L. ^a,b^	*Clavel*	Flowers	Anti-inflammatory, anti-flu, analgesic, anticough, sedative, cardiotonic, and to cure nerves and stomach pain [4,16,20,21].
	**CHENOPODIACEAE**			
38	*Chenopodium album* L. ^b^	*Paico*, *palitaria*, *palitaria blanca*	Branches or buds	To treat blows, dislocations, and sprains [4,21].
39	*Chenopodium ambrosioides* L. ^b^	*Paico*	Whole plant	To treat gallbladder stones and gastrointestinal problems [4,18].
40	*Tradescantia zebrina* Hort. Ex Bosse ^b^	*Hoja de la plata*, *lazo de amor*, *oreja de tigre, zebrina*, *calcha*	Whole plant	To prevent postpartum relapse [4,21].
	**CRASSULACEAE**			
41	*Kalanchoe gastonis* Bonnieri ^b^	*Dulcamara*, *mala madre*	Leaves	To treat general digestive disorders [21].
	**CUCURBITACEAE**			
42	*Cucurbita ficifolia* Bouchè, Verh. ^a,b^	*Alcayata*, *zambo*	Whole plant	To treat blows [4].
43	*Cucurbita maxima* D’uchense ex Lam. ^a,b^	*Zapallo*	Leaves	To cure diarrhea in children from 1 to 6 months of age [4].
44	*Cucurbita pepo* L. ^a,b^	*Sambo*	Latex	To treat general dermatological disorders [21].
	**CUPRESSACEAE**			
45	*Cupressus lusitanica* Mill. ^a,b^	*Ciprés*	Fruits	To control baldness [4].
	**FABACEAE**			
46	*Medicago sativa* L. ^a,b^	*Alfalfa*	Leaves	To treat circulatory problems, especially lack of sensitivity at the body extremities (e.g., hands, feet, and/or toes) [4].
47	*Vicia faba* L. ^a,b^	*Haba*	Leaves	To treat headache [4].
	**GENTIANACEAE**			
48	*Centaurium erythraea* Rafr. ^b^	*Pedorrera*, *canchalagua*	Whole plant	To cure body malaise [4,21,22].
	**GERANIACEAE**			
49	*Erodium* cf. *cicutarium* (L) L’Hér. Ex Aiton ^b^	*Agujilla*, *aujilla*	Branches	It is used orally to treat general disorders and culture-related syndromes [21].
50	*Pelargonium graveolens* L’Hér. ex Aiton. ^a,b^	*Esencia de rosa*	Flowers, leaves, stalks	Anti-inflammatory, analgesic, febrifuge, antidiabetic, antidiarrheal, to treat gallbladder and liver problems, a digestive, to cure gastric ulcers, wounds, burns, respiratory diseases, jaundice, infertility, and urinary stones. It is also used to cure vaginal infections before and after childbirth [4,16,21,22].
51	*Pelargonium odoratissimum* (L.) L’ Hér. ^a,b^	*Malva olorosa*	Flowers, leaves, stalks, branches	Anti-inflammatory, analgesic, carminative and tonic, diuretic, antidiarrheal, and to cure colic, neurological and heart problems, and children’s colds [4,16,20,21,22].
52	*Pelargonium zonale* (L.) L’Hér. ^a^	*Geranio*	Flowers	To cure vaginal infections before and after childbirth [4,22].
	**JUGLANDACEAE**			
53	*Juglans nigra* L. ^a,b^	*Nogal*	Leaves	To cure gastrointestinal and renal-urological problems [18].
	**LAMIACEAE**			
54	*Mentha piperita* L. ^a,b^	*Menta*, *menta negra*	Leaves, stalks, branches	Analgesic, antidiarrheal, anti-flu, anti-inflammatory, antitussive, carminative, digestive, tonic, and against stomach pain, stomach colic, to cure indigestion and the cold; pain relief (joints, head, throat); to treat gastrointestinal, respiratory, skin (inflammation, bruises), and neurological problems [4,18,20,21,22].
55	*Mentha pulegium* L. ^a,b^	*Menta de castilla*	Branches	To treat stomach colic, indigestion, and the cold [4].
56	*Mentha spicata* L. ^a,b^	*Hierba buena*, *menta*, *menta negra*	Leaves	Anti-inflammatory, anti-flu, analgesic, digestive, antitussive, carminative, febrifuge, to cure stomach colic and the cold; pain relief (joints, head, throat); to treat gastrointestinal, respiratory, and renal-urological problems [4,18,20,21,22].
57	*Ocimum basilicum* L. ^a,b^	*Albahaca*, *albahaca blanca*	Flower, leaves	Anti-inflammatory, antispasmodic, anti-flatulence, analgesic, febrifuge, digestive, stimulant of lactation, relaxant, to treat headache, coughs, heart problems, nerves, gastritis, high blood pressure, internal infections, pain relief (joints, head, throat); to treat gastrointestinal and skin (inflammation, bruises) problems [16,18,21].
58	*Origanum majorana* L. ^a,b^	*Mejorana*	Leaves	Pain relief (joints, head, throat) [18,22].
59	*Origanum x majoricum* Camb. ^a,b^	*Orégano*, *oregano de castilla*	Whole plant without roots	To treat digestive problems [21].
60	*Origanum vulgare* L. ^a,b^	*Orégano*	Whole plant	Pain relief (joints, head, throat), and to treat gastrointestinal and renal-urological problems [4,22].
61	*Plectranthus unguentarius* Codd	*Oreganón*, *orégano grande*	Leaves	It is used orally to treat digestive problems [21].
62	*Rosmarinus officinalis* L. ^a,b^	*Romero*	Branches, whole plant	To cure the fever or the cold caused by cold air or strong winds (locally known as mal aire (bad air) ^c^. Pain relief (joints, head, throat); to cure gastrointestinal, skin (inflammation, bruises), and neurological problems [4,18,21,22].
63	*Salvia tiliifolia* Vahl. ^b^	*Santa María*	Whole plant without roots	The plant is rubbed to treat culture-related syndromes [21].
64	*Thymus vulgaris* L. ^a,b^	*Tomillo*	Branches, leaves	To cure indigestion, gastrointestinal, and renal-urological problems; pain relief (joints, head, throat) [4,18,21].
	**LAURACEAE**			
65	*Cinnamomum verum* J. Presl ^b^	*Canela*	Bark	Pain relief (joints, head, throat), and to treat gastrointestinal and respiratory problems [18].
	**LILIACEAE (AMARYLLIDACEAE)**			
66	*Allium sativum* L. ^a,b^	*Ajo*	Garlic, bulbs	To cure coughs; pain relief (joints, head, throat), and to treat gastrointestinal and respiratory problems [4,18].
	**LINACEAE**			
67	*Linum usitatissimum* L. ^a,b^	*Linaza*	Seeds, leaves, stalk	It is used to treat general disorders of the digestive and urological systems [15,22]. Anti–inflammatory, digestive, hepatic, diuretic, to treat stomachache and kidney problems, inflammation of liver and kidney, and gastrointestinal and respiratory problems [4,16,18,20].
	**MALVACEAE**			
68	*Alcea rosea* L. ^a,b^	*Malva goma, malva rosa*	Flowers, bark	To treat liver and kidney pain, and used as a diuretic, analgesic, and depurative [4,20,21].
69	*Corchorus siliquosus* L	*Té*	Whole plant	To treat general digestive disorders [22].
70	*Lavatera arborea* L. ^a^	*Malva*, *puka malva*	Flowers	To treat liver and kidney inflammations [4].
71	*Malva arborea* (L.) Webb & Berthel. ^a^	*Malva altea*, *malva blanca*, *malva alta*	Flowers	Anti-inflammatory, antidiarrheal, febrifuge, depurative, diuretic, tonic, digestive, to treat obesity, constipation, and insect bites [4,21].
72	*Malva parviflora* L. ^a,b^	*Malva blanca*	Branches, flowers	To treat general gynecological and urological disorders [21].
	**MORACEAE**			
73	*Ficus carica* L. ^a,b^	*Higo*, *breva*, *higuera*	Leaves	It is used orally to treat gynecological disorders [21].
	**MYRTACEAE**			
74	*Corymbia citriodora* (Hook.) K.D. Hill & LAS. Johnson ^a,b^	*Eucalipto oloroso*, *eucalipto aromático*	Branches	It is used for inhalations to treat disorders of the respiratory system [21].
75	*Myrtus communis* L. ^b^	*Arrayán*	Leaves, fruits	To treat fever, gastrointestinal, respiratory, and skin (inflammation, bruises) problems [18].
76	*Syzygium aromaticum* L. ^a,b^	*Clavo de olor*	Flower buds, peduncles	Pain relief (joints, head, throat), and to cure gastrointestinal problems [18].
	**OLEACEAE**			
77	*Jasminum grandiflorum* L. ^a,b^	*Jazmín*	Flowers	To treat neurological disorders [22].
	**ONAGRACEAE**			
78	*Fuchsia magellanica* Lam. ^a,b^	*Pena-pena*, *pena*, *pena morada*, *zarcillo*	Flowers	Sedative, disinfectant, wound healer, and relaxant [19,20,21].
	**PINACEAE**			
79	*Pinus radiata* D. Don ^a,b^	*Pino*	Fruits	Against asthma [4].
	PLANTAGINACEAE			
80	*Plantago major* L. ^a,b^	*Llantén*	Whole plant, leaves	Anti-inflammatory, antihemorrhagic, digestive, wound healer, diuretic, to treat liver problems, insomnia, insect bites, liver and kidney inflammation; pain relief (joints, head, throat), and for gastrointestinal, respiratory, skin (inflammation, bruises), renal-urological, and neurological problems [4,18,21,22].
	**POACEAE**			
81	*Cynodon dactylon* (L.) Pers. ^b^	*Grama dulce*, *paja*	Whole plant without roots	It is used orally to treat urological and gynecological disorders [21].
82	*Cymbopogon citratus* (DC.) Stapf. ^a,b^	*Hierba Luisa*, *limonaria*	Leaves	Anti-flatulence, analgesic, digestive, sedative, expectorant, spasmolytic, relaxant and diuretic, anti-inflammatory, to treat high pressure, nerves, gastritis, diarrhea, jaundice, insomnia and the cold, gastrointestinal, respiratory, skin (inflammation, bruises), and neurological problems; pain relief (joints, head, throat) [14,21,22].
83	*Zea mays* L. ^a,b^	*Maíz (pelo de choclo)*	Hair of dried maize, flowers	Against diarrhea and general malaise; pain relief (joints, head, throat), anti-inflammatory; to treat gastrointestinal, respiratory, renal-urological, and neurological problems, skin inflammation, and bruises [4,18,21,22].
	**POLYGONACEAE**			
84	*Rumex obtusifolius* L. ^b^	*Lengua de vaca*, *sacha-gula*	Leaves, flowers	Pain relief (joints, head, throat), and to cure skin inflammation and bruises; anti-inflammatory [18].
	**ROSACEAE**			
85	*Eriobotrya japonica* (Thunb.) Lindl. ^a,b^	*Níspero*, *míspero*, *níspero del japón*	Leaves	It is used orally to treat urinary disorders [21,22].
86	*Poterium sanguisorba* L. ^b^	*Pimpinela*	Whole plant	To treat neurological problems [4].
87	*Rosa x alba* L. ^a,b^	*Rosa blanca*	Flowers	To treat infections and flu [22].
88	*Rosa centifolia* L.	*Rosa roja*	Flowers	To treat neurological problems [4,22].
89	*Rosa cymosa* Tratt. ^a,b^	*Rosa*	Flowers	It is used orally to treat gynecological and urological disorders [21].
90	*Sanguisorba minor* subsp. *Muricata* (Bonnier & Layens) Briq ^a,b^	*Pimpinela*	Leaves	It is used orally to treat neurological problems [21].
	**RUTACEAE**			
91	*Citrus x junos* Siebold ex Tanaka ^a,b^	*Naranja agria*	Fruits	It is used orally to treat dermatological problems [21].
92	*Citrus limetta* Risso ^a,b^	*Lima dulce*	Fruits	To prevent high blood pressure [22].
93	*Citrus x limonum* Risso ^a,b^	*Limón*	Seeds	It is used orally to treat dental pain [22].
94	*Citrus sinensis* (L.) Osbeck. ^a,b^	*Hojas de naranja*	Leaves	Antispasmodic, relaxant, antidiarrheal; used as hair tonic; used to treat insomnia, the cold and kidney problems [16].
95	*Ruta graveolens* L. ^a,b^	*Ruda*	Branches, flowers, whole plant	To treat headaches, bad air ^c^, fainting during childbirth, gastrointestinal and neurological problems; pain relief (joints, head, throat) [4,18,21,22].
	**SIMAROUBACEAE**			
96	*Castela tortuosa* Liebm. ^b^	*Hierba de perro*	Leaves, flowers	Pain relief (joints, head, throat), and to treat gastrointestinal problems [18].
	**TILIACEAE**			
97	*Tilia platyphyllos* Scop. ^a^	*Tilo*	Leaves, flowers	To cure respiratory, neurological, and reproductive diseases; anti-inflammatory [18].
	**URTICACEAE**			
98	*Urtica dioica* L. ^b^	*Ortiga*, *ortiga de monte*	Whole plant	Pain relief (joints, head, throat), and to cure gastrointestinal, neurological diseases [18,21].
99	*Urtica urens* L. ^b^	*Chine*, *chini*, *ortiga común*	Whole plant	To treat intestinal infection and blows [4,21].
	**VALERIANACEAE**			
100	*Valeriana officinalis* L. ^b^	*Valeriana*, *guasilla*	Leaves	To cure gastrointestinal and neurological diseases [4].
	**VERBENACEAE**			
101	*Phyla dulcis* (Trevir.) Moldenke ^a,b^	*Buscapina*	Whole plant	To treat stomachache [22].
102	*Phyla scaberrima* (A. Juss. Ex Pers.) Moldenke ^b^	*Buscapina*, *novalgina*	Whole plant without roots	It is used orally to treat digestive problems [4,21].
	**VIOLACEAE**			
103	*Viola odorata* L. ^a,b^	*Violeta*, *violeta de huerta*, *violeta de jardín*	Flowers	To cure coughs [4,21,22].
104	*Viola tricolor* L. ^b^	*Pensamiento*	Flowers	Analgesic, antidiarrheal, anti-flu, anti-inflammatory, antiseptic, diuretic, febrifuge; to cure the hoarseness and headache; to treat kidney, skin, heart, and nerve problems [4,18,21].
	**ZINGIBERACEAE**			
105	*Hedychium coronarium* J.Köning ^a,b^	*Jazmín de río*, *caña agria*	Stems	It is used orally to treat problems of the urinary system [21,22].

^a^ The plant is also cultivated. ^b^ The phytochemistry and biological activities of the plant have already been investigated by scholars working in countries other than Ecuador. ^c^ A supernatural disease.

The fact that Asteraceae (Compositae) is the family with the highest number of medicinal taxa is not unexpected because it is one of the largest flowering plant families, consisting of over 32,000 known species in over 1900 genera distributed worldwide [23,24]. All species are good sources of inulin, a natural polysaccharide with strong prebiotic properties. They have also demonstrated high antioxidant, anti-inflammatory, and antimicrobial activities, as well as diuretic and wound-healing properties. A few taxa also contain cytotoxic metabolites. These pharmacological effects are attributed to a range of phytochemical compounds, including polyphenols, phenolic acids, flavonoids, polyenes, alkaloids, sesquiterpene lactones, diterpenoids, triterpenes, and essential oils [77]. Species belonging to Lamiaceae are known for the contents of aromatic volatile compounds, whereas the characteristic chemical constituents of Solanaceae species are biologically active alkaloids of the steroidal, tropane, and nicotine types [77].

Some species belonging to the Lycopodiaceae family are traditionally used to treat supernatural diseases and to perform religious rituals due to their psychoactive effects [78]. The extracts contain alkaloids of the *Lycopodium* type and exhibited an interesting cholinesterase activity [3]. Therefore, related Lycopodiaceae species reported in Table 1, such as *H. sellifolia*, *L. weberbaueri*, and *H. austroecuadorica* deserve to be studied from a phytochemical and pharmacological point of view, especially in the search for natural remedies for age-related neurodegenerative diseases [79]. In this context, it is worthwhile to note that a few endemic species belonging to the genus *Fuchsia*, such as *F. harlingii*, *F. hypoleuca*, and *F. loxensis*, which are used in the traditional medicine for neurological treatments, have not yet received adequate scientific attention by scholars of natural products.

Other still uninvestigated native plants which might offer interesting research opportunities belong, inter alia, to the families of Asteraceae, Fabaceae, Ericaceae, Orchidaceae, Piperaceae, and Solanaceae, which are well known sources of specialized metabolites with various chemical structures and different biological activities [77].

On the other hand, several species of the genus *Amaranthus* are traditionally cultivated in Central and South America, where local people use seeds or leaves as food and herbal remedies [80]. Therefore, the traditional uses of *A. caudatus*, *A. cruentus*, *A. hybridus*, and *A. quitensis* deserve to be validated with scientific evidence to enhance their sustainable use as a food supplement or in phytopharmaceutical products. Another plant of promising scientific and practical interest is *Phyla strigulosa* (family Verbenaceae). In fact, in preliminary investigations, we have found that it can be used to prepare non-caloric sweeteners.

The plants reported in Table 1 and Table 2 are most widely used as analgesic, antidiarrheal, anti-flu, anti-inflammatory, antitussive, carminative, sedative, digestive, tonic, and pain relief (joints, head, throat, stomach) remedies, against colic, to cure the cold, and to treat gastrointestinal, respiratory, dermatological, renal-urological, gynecological, and neurological problems. The frequency of these uses clearly reflects the spread of these diseases in Indigenous communities. In this regard, it is important to highlight the limited number of plants used against cancer, while it is quite stunning to note the large number of species used to cure nervous and general neurological problems. It would be interesting to investigate the causes of such diseases in a relatively poor country such as Ecuador, because these diseases are usually considered typical of affluent societies.

Several plants listed in Table 1 and Table 2, which belong to different genera and even to different families, are often used to treat the same disease or the same group of diseases. This finding may suggest that compounds with different chemical structures display the same bioactivity or that compounds of the same type occur in the different species.

Most plants reported in this review are used against well-defined pathologies, for which appropriate in vitro biological tests and even clinical trials can be executed to confirm the effectiveness of the positive effects and to direct the isolation of bioactive compounds. Other plants are, instead, used against ill-defined diseases, such as those employed to cure ‘culture-related syndromes’, or ‘a restless and confused child’, or a generic ‘disease of the body’. Even harder to decipher, under the perspective of western medicine, are the so-called supernatural and magical diseases such as the ‘mal aire (bad air)’, ‘air water’ or ‘evil eye’. However, these beliefs are part of the cultural heritage of this population and are, therefore, of great anthropological interest.

## 4. Conclusions

We believe that a critical evaluation of the ethnobotanical and ethnopharmacological information contained in this review may give several opportunities to develop innovative research and to design practical applications of several traditional plants of Ecuador, with benefits not only to the Indigenous communities but to the entire population of the country. Introduced medicinal plants (Table 2), whose chemical components and biological activities are usually known, have the potential of immediate practical applications. *Allium sativum*, *Mentha piperita*, and *Aloe vera* are representative examples of plants with these characteristics. On the other hand, endemic medicinal species are of primary importance for Ecuador, which is the only owner in the world of unique botanical resources that must therefore be preserved with extreme care. Moreover, the phytochemistry and biological activities of little-investigated endemic plants deserve to be investigated for their potential as new natural sources of isolated compounds or extracts with therapeutic interest. Examples of plants endemic to Ecuador, which have already aroused great scientific interest, are: *Lepichinia mutica* Benth. (Lamiaceae), which produces appreciable amounts of carnosol, a compound with potent anti-BuChE activity [81]; *Gynoxys miniphylla* Cuatrec. (Asteraceae), whose EO exhibits promising cholinergic, antiviral, and analgesic effects [82], and *Clinopodium tomentosum* (Kunth) Govaerts (Lamiaceae), whose leaf extract influences in vitro cell proliferation and angiogenesis on primary cultures of porcine aortic endothelial cells [83].

## Data Availability

Not applicable.
